# Recurrent Severe Infections During Teclistamab Therapy in a Kidney Transplant Recipient With Relapsed Multiple Myeloma: A Case Report

**DOI:** 10.7759/cureus.109924

**Published:** 2026-05-30

**Authors:** Maxime Rousseau, Anne Deweweire, Julien Depaus, Géraldine Claes

**Affiliations:** 1 Hematology, Institut Jules Bordet, Mons, BEL; 2 Hematology, Centre Hospitalier EpiCURA, Hornu, BEL

**Keywords:** bispecific antibody, infections in immunocompromised, kidney transplant recipient, relapsed and refractory multiple myeloma, teclistamab

## Abstract

Bispecific antibodies targeting B-cell maturation antigen (BCMA), such as teclistamab, have significantly improved outcomes in relapsed/refractory (R/R) multiple myeloma (MM) but are associated with an increased risk of infections and hypogammaglobulinemia. We report the case of an 82-year-old kidney transplant recipient with penta-refractory IgG kappa MM treated with teclistamab. Despite a rapid hematological response, the patient developed multiple severe infections, predominantly respiratory, in the context of profound hypogammaglobulinemia and pre-existing bronchiectasis. Immunoglobulin (Ig) replacement therapy and azithromycin prophylaxis were introduced, leading to a marked reduction in infectious episodes while maintaining myeloma control and stable graft function initially. Due to the recurrent occurrence of these respiratory infections, the administration of teclistamab was also spaced out, which led not only to a reduction in the occurrence of these infections but also to a relapse of MM. This case illustrates the challenges associated with managing solid organ transplant recipients receiving teclistamab for MM, particularly with regard to infectious complications and severe immunosuppression in these patients. The challenge lies in striking a balance between reducing the risk of infection and maintaining the best possible hematological response.

## Introduction

Bispecific antibody therapies are an integral part of the new therapeutic arsenal for the management of multiple myeloma (MM) and have emerged as a major therapeutic advance in heavily pretreated MM. Teclistamab, a bispecific antibody targeting B-cell maturation antigen (BCMA), which is a protein that is overexpressed on the surface of malignant plasma cells in MM, demonstrated superiority in terms of survival following the pivotal MajesTEC-1 trial, showing gains in progression-free survival (PFS) and overall survival (OS) in relapsed/refractory (R/R) MM patients [1-3]. However, these treatments are associated with an increased risk of infection, linked to hypogammaglobulinemia and immune dysregulation [4,5].

Solid-organ transplant recipients represent a particularly vulnerable population because of chronic immunosuppression and impaired cellular immunity. These patients are typically excluded from clinical trials evaluating novel immunotherapies, leaving limited evidence to guide clinical decision-making. The safety of BCMA-targeted bispecific antibodies in this setting remains poorly documented.

We describe an 82-year-old kidney transplant recipient with R/R MM treated with teclistamab who developed recurrent severe infections requiring repeated hospitalizations. This case illustrates the challenges of balancing the effectiveness of myeloma treatment and the risk of infection in a patient with severe immunodeficiency. This raises the question of optimizing anti-infective prophylaxis and immunoglobulin (Ig) substitution in these already frail patients.

## Case presentation

An 82-year-old man was diagnosed with IgG kappa monoclonal gammopathy of undetermined significance (MGUS) in 2000, progressing to smoldering MM (SMM) in 2006, and symptomatic MM in 2012. In 2011, he underwent a kidney transplantation for chronic interstitial nephropathy and nephroangiosclerosis. At this time, there are no signs of myeloma-related kidney damage. Maintenance immunosuppression consisted of a calcineurin inhibitor, tacrolimus, with stable graft function over time.

**Figure 1:**
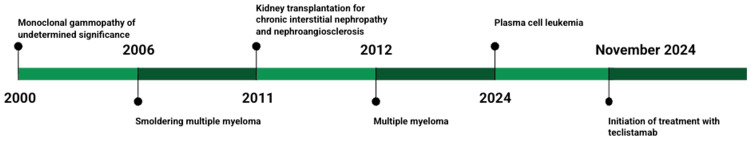
Timeline of the patient’s key diagnoses and treatments from the diagnosis of monoclonal gammopathy of undetermined significance to the initiation of treatment with teclistamab

Between 2012 and 2024, he received multiple lines of MM therapy according to conventional protocols and guidelines from recent years, including a proteasome inhibitor, immunomodulatory agents, corticosteroids, and an anti-CD38 monoclonal antibody (daratumumab). He eventually became penta-refractory. In 2024, relapse was characterized by circulating plasma cells consistent with secondary plasma cell leukemia. Teclistamab was initiated in November 2024 (Figure 1). Treatment was initially well tolerated, without high-grade cytokine release syndrome (CRS) or immune effector cell-associated neurotoxicity (ICANS). A rapid hematological response was observed, including clearance of circulating plasma cells and a marked reduction in monoclonal protein. Complete remission was achieved according to the International Myeloma Working Group (IMWG) criteria, and the kidney transplant had not deteriorated [6].

Within months of treatment initiation, the patient developed recurrent infections requiring hospitalization (Table 1). Thoracic imaging revealed bronchiectasis, likely contributing to susceptibility to infection.

**Table 1:** List of infections that occurred during treatment with teclistamab and the treatments used

Cycle of teclistamab	Infection	Treatment
Cycle 1 (11/2024)	Lower-limb cellulitis	Vancomycin, cefepim, amoxicillin-clavulanate
Cycle 2 (12/2024)	SARS-CoV-2 pneumonia	Remdesivir
Cycle 3 (01/2025)	*Proteus mirabilis* pneumonia	Cefepim
Cycle 4 (02/2025)	Pneumonia - no microbiological documentation	Meropenem, piperacillin-tazobactam
Cycle 5 (1) (03/2025)	Pneumonia - no microbiological documentation	Meropenem
Cycle 5 (2) (03/2025)	Pneumonia - no microbiological documentation	Meropenem

During teclistamab therapy, progressive hypogammaglobulinemia was documented, with levels falling to as low as 1 g/L of IgG. Ig replacement therapy was initiated during the first lines of treatment for MM and subsequently intensified because of persistently low IgG levels and ongoing infections during treatment with teclistamab. In the infections described above, serum IgG levels were consistently < 4 g/L, despite Ig replacement therapy, which was therefore gradually increased. The regimen was adjusted to maintain serum IgG concentrations above 7 g/L, as recommended during treatment with teclistamab. Azithromycin prophylaxis was introduced in view of recurrent respiratory infections and bronchiectasis. Throughout all treatment lines, the patient received prophylactic treatment against infection with acyclovir and sulfamethoxazole-trimethoprim.

Following these interventions, the frequency and severity of infections decreased markedly, particularly after serum IgG levels reached 7 g/l. No further hospitalizations related to infection occurred for several months. The response to myeloma was initially maintained. Subsequently, new infections developed despite a serum IgG level of > 7 g/L, and it was decided to space out teclistamab administrations in order to reduce the infectious toxicity of the treatment, which led to a recurrence of the disease. The patient eventually died as a result of the progression of the disease and the onset of new respiratory infections.

## Discussion

This case illustrates the cumulative immunosuppressive burden resulting from advanced age, heavily pretreated MM, chronic transplant-related immunosuppression, and BCMA-targeted bispecific therapy. Each of these factors independently increases susceptibility to infection, and their combination likely resulted in profound immune vulnerability. Data on solid organ transplant patients treated with bispecific antibodies for MM are scarce, with only a few cases reported in the literature. Solid-organ transplant recipients are systematically underrepresented in trials of T-cell-redirecting therapies.

Solid organ transplant recipients are intrinsically at higher risk of both malignancy and infection due to long-term immunosuppression, most often with calcineurin inhibitors (CI) and mycophenolate mofetil (MMF). CI reduces the proliferation of T lymphocytes (cellular immunity) [7], and MMF reduces the proliferation of B and T lymphocytes, as well as the antibody production (cellular and humoral immunity) [8].

Patients with MM are immunosuppressed due to the nature of the disease and the multiple treatments associated with it (immunomodulators, proteasome inhibitors, corticosteroids, and daratumumab) [9]. Bispecific antibodies targeting BCMA, such as teclistamab, target BCMA on plasma cells and CD3 on T cells, forming an immunological synapse that leads to T cell activation and tumor cell lysis [10], inducing profound B-cell depletion and hypogammaglobulinemia.

In different clinical trials and reviews, infections are frequent in patients treated with teclistamab, with mainly respiratory infections, and more than half of patients require hospitalization. These infections led to treatment interruption (13%), a 14-day delay in administration (30%), and dose spacing or reduction (1%). The majority of these infections occurred in patients with hypogammaglobulinemia <400 mg/dL at the time of infection [11]. Hypogammaglobulinemia was frequently observed [1] and is a major risk factor for infections secondary to bispecific antibodies.

In our patient, severe recurrent respiratory infections occurred in the setting of profound hypogammaglobulinemia and pre-existing bronchiectasis. Introduction of Ig replacement therapy (IVIg) and antimicrobial prophylaxis was associated with a reduction in infectious burden while preserving initial disease control. However, subsequent spacing of teclistamab dosing in response to infectious complications coincided with biochemical relapse of MM, illustrating the narrow therapeutic window between infection risk and disease control in this context.

Current expert recommendations suggest regular monitoring of Ig levels and early intravenous Ig replacement in patients receiving BCMA-targeted bispecific antibodies, although evidence remains limited and data are not sufficiently mature to allow strong recommendations. They recommend monthly IVIg treatment for the duration of treatment until Ig levels are ≥ 400 mg/dl. For teclistamab, it is recommended that IgG levels be maintained above 700 mg/dl [12]. Antiviral and Pneumocystis prophylaxis are widely recommended, whereas antibacterial prophylaxis remains controversial and generally reserved for selected high-risk patients.

Importantly, this case suggests that standard prophylactic strategies used in relapsed MM may be insufficient in solid organ transplant recipients, who represent an exceptionally immunocompromised population. In such patients, earlier Ig replacement, individualized antimicrobial strategies, and close multidisciplinary collaboration between hematology and transplant teams regarding the management of immunosuppression appear essential.

Finally, the optimal management of baseline transplant immunosuppression during BCMA-targeted therapy remains undefined and should be carefully individualized to balance infectious risk against graft preservation and oncologic control.

## Conclusions

This case highlights the profound cumulative immunosuppressive burden in a kidney transplant recipient with MM treated with teclistamab, resulting in severe infectious complications. This underscores the importance of early Ig replacement therapy, as well as systematic and personalized antimicrobial prophylaxis, in this high-risk population. The routine use of antibiotics, even before treatment begins, is open to question. Management should rely on close multidisciplinary collaboration to balance infectious risk, graft preservation, and myeloma control.
